# Proline, hydroxyproline, and pyrrolidone carboxylic acid derivatives as highly efficient but reversible transdermal permeation enhancers

**DOI:** 10.1038/s41598-022-24108-6

**Published:** 2022-11-14

**Authors:** Monika Kopečná, Miloslav Macháček, Jaroslav Roh, Kateřina Vávrová

**Affiliations:** 1 Skin Barrier Research Group, Charles University, Faculty of Pharmacy in Hradec Králové, Akademika Heyrovského 1203, 50005 Hradec Králové, Czech Republic; 2 Department of Biochemical Sciences, Charles University, Faculty of Pharmacy in Hradec Králové, Akademika Heyrovského 1203, 50005 Hradec Králové, Czech Republic; 3 Department of Organic and Bioorganic Chemistry, Charles University, Faculty of Pharmacy in Hradec Králové, Akademika Heyrovského 1203, 50005 Hradec Králové, Czech Republic

**Keywords:** Drug delivery, Medicinal chemistry, Pharmaceutics

## Abstract

Overcoming the skin barrier properties efficiently, temporarily, and safely for successful transdermal drug delivery remains a challenge. We synthesized three series of potential skin permeation enhancers derived from natural amino acid derivatives proline, 4-hydroxyproline, and pyrrolidone carboxylic acid, which is a component of natural moisturizing factor. Permeation studies using in vitro human skin identified dodecyl prolinates with *N-*acetyl, propionyl, and butyryl chains (Pro2, Pro3, and Pro4, respectively) as potent enhancers for model drugs theophylline and diclofenac. The proline derivatives were generally more active than 4-hydroxyprolines and pyrrolidone carboxylic acid derivatives. Pro2–4 had acceptable in vitro toxicities on 3T3 fibroblast and HaCaT cell lines with IC_50_ values in tens of µM. Infrared spectroscopy using the human *stratum corneum* revealed that these enhancers preferentially interacted with the skin barrier lipids and decreased the overall chain order without causing lipid extraction, while their effects on the *stratum corneum* protein structures were negligible. The impacts of Pro3 and Pro4 on an in vitro transepidermal water loss and skin electrical impedance were fully reversible. Thus, proline derivatives Pro3 and Pro4 have an advantageous combination of high enhancing potency, low cellular toxicity, and reversible action, which is important for their potential in vivo use as the skin barrier would quickly recover after the drug/enhancer administration is terminated.

## Introduction

Transdermal drug delivery represents an advantageous alternative to conventional drug administration routes^1,2^. During the past few decades, drug delivery through the skin evolved from an abstract idea to a real part of the pharmaceutical market. Administration of a broader palette of drugs is mainly hampered by the uppermost skin layer, the *stratum corneum*, which evolved to protect the organism from excessive water loss and entry of exogenous agents^3^. One approach to temporarily decrease the skin barrier properties uses penetration enhancers, also called permeation enhancers or absorption promoters^1,2,4–7^. These chemical compounds can interact with the *stratum corneum* extracellular lipids and proteins or promote drug partitioning from the formulation into the skin. Although hundreds of enhancers have been identified to date, versatile enhancers with high activity, low toxicity, and a well-defined mechanism of action are scarce^6^.

The design of new enhancers often aims at amphiphilic structures as they would likely target the principal permeation pathway through the *stratum corneum* lipids. Placing a biodegradable bond, for example, an ester, between the polar head and the lipophilic tail of such amphiphiles significantly reduces the enhancer’s half-life, which can minimize its toxicity and ensure reversibility of its action. Another approach to lower enhancer toxicity exploits natural compounds^5,7^. Natural compounds have successfully been used as the polar heads of amphiphilic enhancers (such as α- and ω-amino acids^8–16^ and sugars) or their lipophilic tails (e.g., terpene moieties)^17^.

**Figure 1:**
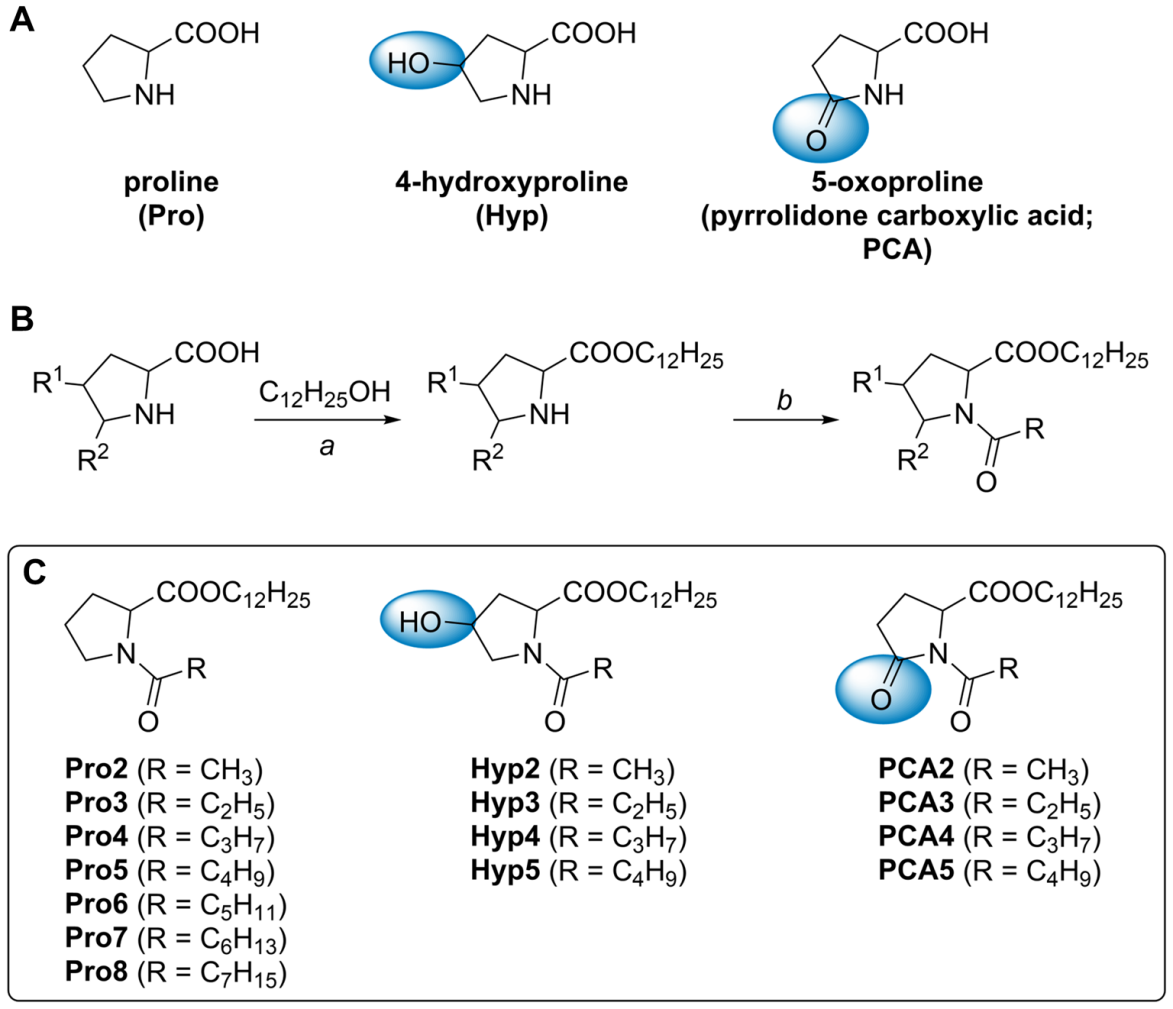
Parent amino acids Pro, Hyp and PCA (**A**), synthesis (**B**) and structures (**C**) of the studied enhancers. a—HCl(g)/120 °C/7 h for Pro; HCl(g)/70 °C/7 h for Hyp; and *N,N*′-dicyclohexylcarbodiimide (DCC)/4-dimethylaminopyridine (DMAP)/CH_2_Cl_2_/0 °C to room temperature (rt)/overnight for PCA. *b*—RCOOH/DCC/DMAP/CHCl_3_/0 °C to rt/overnight for Pro2–8 and Hyp2–5; RCOCl/diisopropylethylamine/toluene/rt/overnight for PCA3–5; Ac_2_O/DMAP/CHCl_3_/rt/5 h for PCA2.

Several previous studies of amino acid-based enhancers identified proline (Pro) derivatives^8–10,15^, in particular, *N-*acetyl proline dodecyl ester (Pro2^15^, Fig. 1) as potent and reversible broad-spectrum enhancers with low toxicity in vitro and in vivo. Notably, no enantioselectivity in enhancer action was observed^15,18^. Here, we explore the skin permeation-enhancing potential of proline-derived amphiphiles in two ways (Fig. 1): (1) probing the *N-*acyl chain length and (2) changing the pyrrolidine ring substitution. The rationale behind changing the *N-*acyl chain length stems from our previous studies on short-chain ceramide analogs, where *N-*butanoyl and *N*-hexanoyl sphingosines markedly increased the permeability of skin lipid models and human *stratum corneum* (more than *N*-acetyl sphingosine)^19^. The modifications of the pyrrolidine ring involved two natural and non-toxic compounds with potentially beneficial actions in the skin: 4-hydroxyproline (Hyp) and pyrrolidone carboxylic acid (PCA). Hyp is a principal constituent of collagen, and its derivative *N-*acetyl-Hyp (oxaceprol) is an established drug for managing osteoarthritis with atypical anti-inflammatory action. PCA (5-oxoproline, pyroglutamic or pidolic acid) is a component of a natural moisturizing factor formed by filaggrin hydrolysis and glutamate (glutamine) cyclization.

Our work aimed to prepare and study three series of potential enhancers – derivatives of Pro, Hyp, and PCA (all were used as racemates). The carboxyl groups were converted to dodecyl esters, and the nitrogens were modified with acyls with 2–8 carbons (Fig. 1). The permeation-enhancing potencies of the prepared compounds were examined for model drugs theophylline (TH) and diclofenac (DC) using human skin in vitro^8,10^. The in vitro toxicities of selected enhancers were assessed on HaCaT keratinocyte and 3T3 fibroblast cell lines, and the enhancers’ modes of action were investigated using infrared spectroscopy on isolated human *stratum corneum*.

## Results and discussion

### Synthesis

The starting amino acids Pro, Hyp, and PCA (racemic) were converted to their dodecyl esters and then *N-*acylated to yield the final products Pro2–8, Hyp2–5, and PCA2–5, respectively (Fig. 1). The number in the compound code refers to the *N*-acyl chain length. First, dodecyl esters were prepared by esterification as this procedure is inexpensive, and most amino acid esters can be obtained by simple crystallization^15,20^, but the yields of Pro and Hyp dodecyl esters were ~ 20% (and Pro dodecyl ester still required column chromatography). An acceptable yield (84%) of PCA dodecyl ester was obtained using carbodiimide. Pro2–8 and Hyp2–5 were then obtained using carbodiimide coupling in 84–95% yields. PCA2 was prepared using acetic anhydride in 91% yield, while acyl chlorides were used for PCA3–5 in 73–77% yields.

### Effects of Pro, Hyp, and PCA derivatives on the flux and skin retention of theophylline (TH)

First, we used 5% TH as a model permeant representing drugs of small size (180 g/mol) and balanced lipophilicity (logP ~ 0). The prepared enhancers were applied at 1% in 60% aqueous propylene glycol (PG) to human skin in vitro; 60% PG was used as a negative control. PG was selected for its synergy with amphiphilic enhancers^15^. Each model drug was co-applied with the enhancer at infinite dose. Notably, pretreatment experiments (1% enhancer for 24 h, followed by the drug) did not yield any considerable enhancement of drug flux, likely due to a temporary enhancing action; see below.

**Figure 2:**
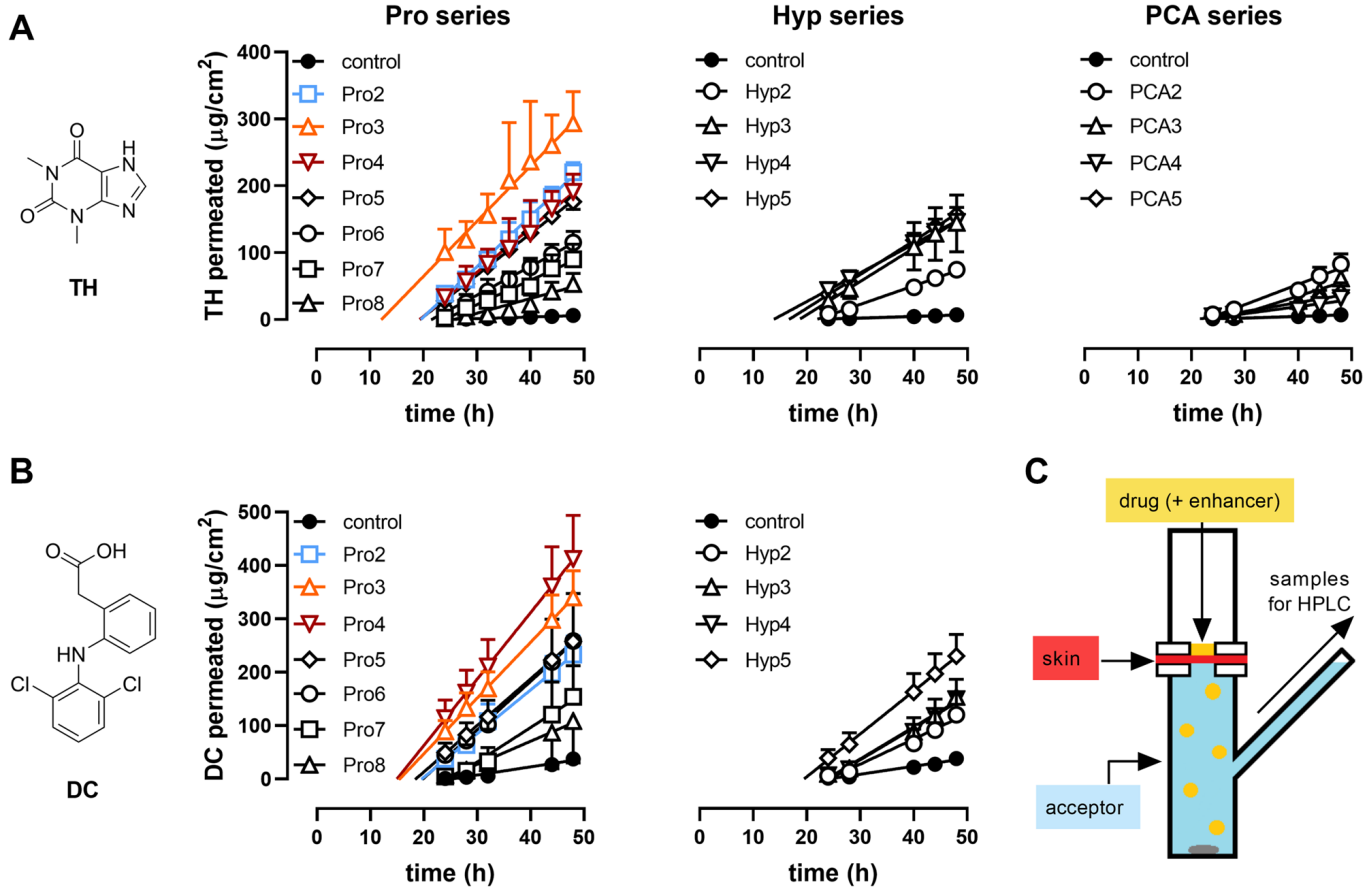
The effects of the studied Pro, Hyp and PCA derivatives on the permeation of the model drugs (infinite dose) theophylline (TH, applied as 5% suspension in 60% PG, **A**) and diclofenac (DC, applied as 3% solution in 60% PG, **B**) through human skin, and the scheme of modified Franz diffusing cell used in the experiments (**C**). Data are presented as the means ± SEM; n ≥ 3. For flux values and statistical significance, see Table 1.

The control sample, TH without the tested compounds, yielded a flux of 0.27 ± 0.06 µg/cm^2^/h (Fig. 2, Table 1), which corresponds to the permeability coefficient of 1.1 × 10^–5^ cm/h and is comparable to previous studies^17,21^. Compounds Pro2–7 significantly increased TH flux with a maximum at Pro3 (8.16 ± 0.81 µg/cm^2^/h); the Pro8 effect was not statistically significant. The potencies of Pro2, Pro3, and Pro4 were comparable, with the enhancement ratios (ERs, fold increase in flux over control) of 28, 31, and 25, respectively. The Pro2 effect was comparable to the previously described effects of its enantiomers L-Pro2 and D-Pro2 (ER ~ 40 on porcine skin)^15^. In the Hyp series, Hyp3 and Hyp5 increased the TH flux approximately 18 and 19-fold (4.84 ± 0.75 and 4.94 ± 0.74 µg/cm^2^/h) over control. In the PCA series, only PCA2 significantly enhanced TH flux (3.04 ± 0.58 µg/cm^2^/h, ER = 12), and further *N-*acyl prolongation diminished their enhancing potencies. The decrease in enhancing activity in the PCA derivatives compared to Pro derivatives is consistent with a decline in ER from 46 to 13 for hydrocortisone upon introducing carbonyl in pyrrolidine enhancers in hairless mouse skin^22^.

**Table 1:** Effects of Pro, Hyp, and PCA derivatives on the flux and skin retention of model drugs theophylline and diclofenac.

Compd	Theophylline (TH; 5%)				Diclofenac (DC; 3%)			
	*J*_*SS*_ (µg/cm^2^/h)	*ER*	*C*_*EPIDERMIS*_ (µg/mg)	*C*_*DERMIS*_ (µg/mg)	*J*_*SS*_ (µg/cm^2^/h)	*ER*	*C*_*EPIDERMIS*_ (µg/mg)	*C*_*DERMIS*_ (µg/mg)
–	0.27 ± 0.06	-	1.88 ± 0.27	0.07 ± 0.01	1.56 ± 0.49	-	5.02 ± 0.87	0.42 ± 0.12
Pro2	7.41 ± 0.39*	28	1.04 ± 0.11	0.37 ± 0.02*	8.22 ± 0.85*	5	7.16 ± 0.79	1.08 ± 0.09
Pro3	8.16 ± 0.81*	31	1.11 ± 0.17	0.32 ± 0.03*	10.41 ± 1.25*	7	11.18 ± 2.39*	1.54 ± 0.28*
Pro4	6.66 ± 0.63*	25	0.83 ± 0.08	0.25 ± 0.01*	12.39 ± 2.09*	8	12.70 ± 2.71*	1.71 ± 0.31*
Pro5	6.25 ± 0.66*	24	0.83 ± 0.12	0.26 ± 0.02*	8.76 ± 1.19*	6	8.51 ± 1.39	1.30 ± 0.25*
Pro6	4.32 ± 0.58*	16	1.14 ± 0.19	0.22 ± 0.03	9.19 ± 2.71*	6	10.26 ± 0.38	1.44 ± 0.31*
Pro7	3.86 ± 0.91*	15	0.95 ± 0.09	0.26 ± 0.04*	7.03 ± 4.64	5	8.63 ± 3.11	1.30 ± 0.75
Pro8	2.27 ± 0.56	9	0.89 ± 0.25	0.19 ± 0.02	4.72 ± 3.12	3	10.20 ± 0.96	1.08 ± 0.28
Hyp2	2.78 ± 0.40*	10	2.87 ± 0.45	0.20 ± 0.03	4.88 ± 1.53	3	9.22 ± 0.32	0.90 ± 0.08
Hyp3	4.84 ± 0.75*	18	3.64 ± 0.85*	0.25 ± 0.07	6.30 ± 1.06	4	9.18 ± 0.62	0.97 ± 0.07
Hyp4	4.27 ± 0.95*	16	1.96 ± 0.56	0.39 ± 0.07*	5.94 ± 0.26	4	8.99 ± 1.23	1.01 ± 0.05
Hyp5	4.94 ± 0.74*	19	1.45 ± 0.41	0.23 ± 0.03	7.84 ± 1.06*	5	9.84 ± 1.22	1.02 ± 0.11
PCA2	3.04 ± 0.58*	12	2.33 ± 0.26	0.22 ± 0.03	n.d	n.d	n.d	n.d
PCA3	2.46 ± 0.58	9	2.99 ± 0.37	0.28 ± 0.04*	n.d	n.d	n.d	n.d
PCA4	1.25 ± 0.09	5	2.53 ± 0.23	0.30 ± 0.14*	n.d	n.d	n.d	n.d
PCA5	1.34 ± 0.56	5	2.14 ± 0.44	0.23 ± 0.07	n.d	n.d	n.d	n.d

*J*_*SS*_, steady-state flux; ER, enhancement ratio; *C*_*EPIDERMIS*_ and *C*_*DERMIS*_, drug concentration in epidermis and dermis, respectively. Enhancers (1%) were co-applied with the respective drug (infinite dose) in 60% PG. Controls were samples without enhancers. Data are presented as means ± SEM; n ≥ 3. * significant compared to the control (without enhancer) at p < 0.05.

Most studied compounds did not influence the epidermal TH concentrations (except for Hyp3, ER = 1.9), whereas the dermal TH levels were 3–fivefold increased by Pro2–5 and 7, Hyp4, and PCA3–4, in a roughly proportional manner to the flux values. None of the enhancers changed the TH solubility in the donor sample (approximately 25 mg/ml). Thus, the Pro, Hyp, and PCA-based enhancers affected TH flux without significantly changing its epidermal retention.

### Effects of Pro and Hyp derivatives on the flux and skin retention of diclofenac (DC)

DC was selected as the second model drug to probe the permeation-enhancing effects of the studied compounds. DC is larger than TH (296 g/mol), and its free acid form is quite lipophilic (logP ~ 4.5). In the *stratum corneum*, which has a pH ~ 5, DC would comprise both the ionized and unionized form (pKa ~ 4.1), whereas almost complete ionization can be expected at pH 7.4 in deeper tissues or the acceptor buffer. Without the studied compounds, DC (3% in 60% PG) flux through human skin was 1.56 ± 0.49 µg/cm^2^/h (Table 1 and Fig. 2), and the permeability coefficient of 5.2 × 10^–5^ cm/h. This permeability coefficient is comparable to previous studies (2.2 × 10^–5^ cm/h for 5% DC in PBS/isopropyl alcohol 9:1 on human skin^23^ or 11.0 × 10^–5^ for 4.9% DC in ethanol/glycerol/water 6:1:3 on rat skin^24^). Pro2–6 significantly raised DC flux (ERs 5–8) with a maximum of 12.39 ± 2.09 µg/cm^2^/h at Pro4. Pro4 also increased DC retention in epidermis and dermis by 3- and fourfold over control. In the Hyp series, only Hyp5 significantly increased DC permeation (7.84 ± 1.06 µg/cm^2^/h) over control. Hyp2–5 did not influence the epidermal and dermal DC concentrations.

Taken together, modifying the pyrrolidine ring of Pro by introducing hydroxyl in Hyp or carbonyl in PCA derivatives did not significantly improve their permeation-enhancing potencies, although Hyp5 also showed enhancing activities worth further studies. Prolongation of the acyl chain in Pro2 to 3 and 4 carbons in Pro3 and Pro4 improved their enhancing potencies for TH and DC model drugs. Thus, we selected Pro3–4 (and Pro2, previously studied as L-enantiomer) for further investigation.

### Cellular toxicities of selected enhancers

**Figure 3:**
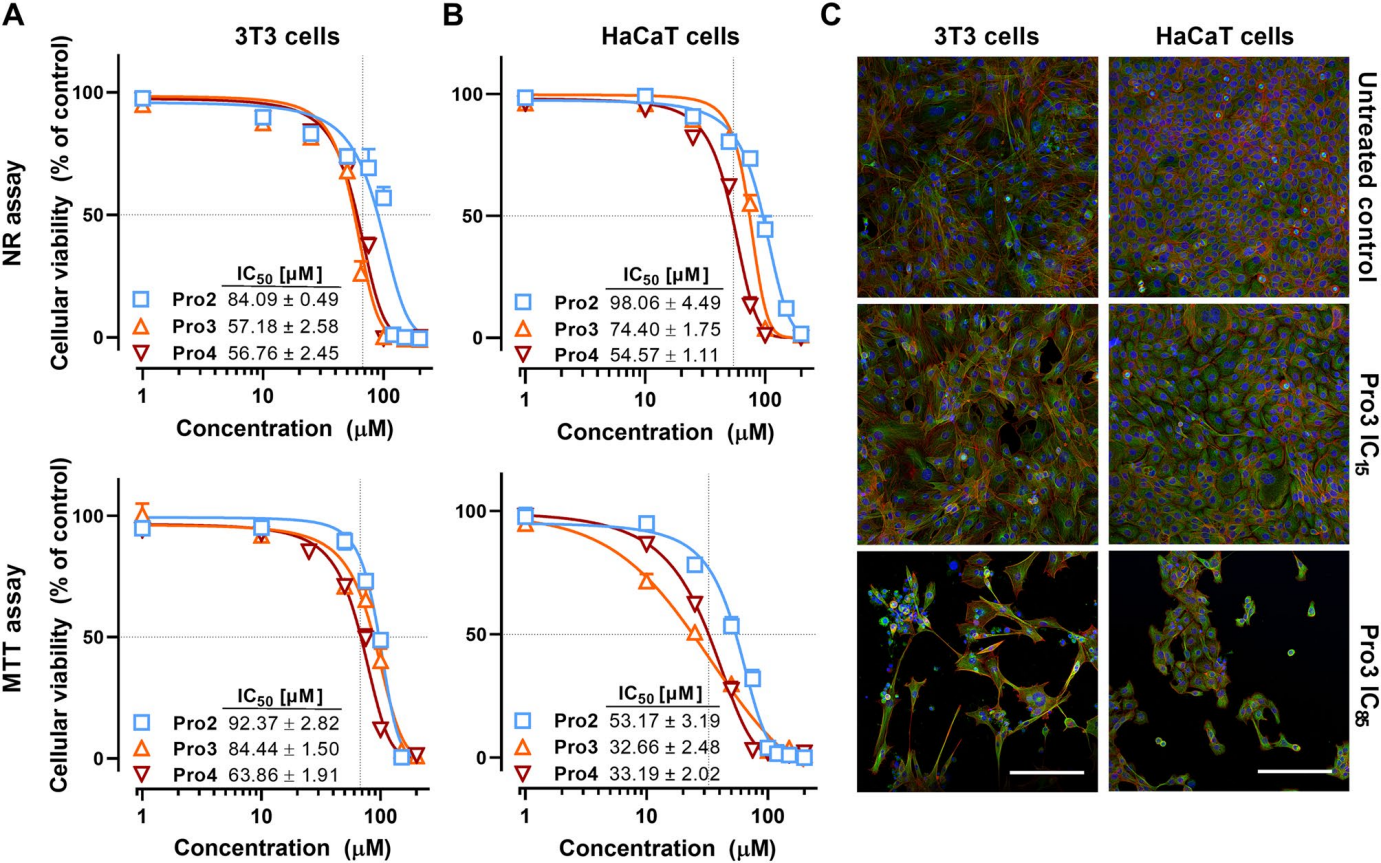
The viability of mouse fibroblast (3T3) and human keratinocyte (HaCaT) cell lines (**A, B**, respectively) determined using NR and MTT assays after 48 h of incubation with the selected enhancers Pro2, Pro3, and Pro4. Data are presented as the means ± SEM; n ≥ 3. (**C**) Morphological changes of 3T3 and HaCaT cells treated with low (IC_15_) and high (IC_85_) doses of Pro3 (others shown in Supplementary Figure S1). Cells were stained for nuclei (blue), microfilaments (green), and microtubules (red). The bar represents 200 µm.

Next, the in vitro toxicities of the most promising enhancers Pro3 and Pro4, along with the parent Pro2, on spontaneously immortalized human keratinocytes (HaCaT) and Swiss albino mouse embryonic fibroblasts (3T3) were studied (Fig. 3). The concentrations causing a 50% decrease in cell viability, the IC_50_ values, after a 48-h incubation of HaCaT cells with the enhancers using the neutral red (NR) uptake assay, which is based on its accumulation in the lysosomes of living cells, were 98.06 ± 4.49, 74.40 ± 1.75, and 54.57 ± 1.11 µM for Pro2, Pro3, and Pro4, respectively. Similar IC_50_ values were found in 3T3 cells (84.09 ± 0.49, 57.18 ± 2.58, 56.76 ± 2.45 µmol/l for Pro2, Pro3, and Pro4, respectively). The cellular toxicities were further probed using the 3-(4,5-dimethylthiazol-2-yl)-2,5-diphenyltetrazolium bromide (MTT) uptake assay, based on the transformation of yellow MTT into purple formazan by mitochondrial reductases of living cells. The MTT assay also yielded toxicity values in tens of µM with trends to higher toxicity with a longer acyl chain length (thus also higher lipophilicity) of enhancers (Fig. 3).

These Pro2–4 intrinsic toxicity values are lower than the toxicities of previously studied sugar derivative 12Glc6N (around 20–25 µM on HaCaT and 3T3 cells)^21^ and comparable to potent amino acid-terpene derivative B-DAK (IC_50_ around 50 µM)^17^. Considering that another amino acid-based enhancer Transkarbam 12 (with the in vitro cellular toxicities around 21–26 µM on HaCaT and 3T3 cells^11^) had low in vivo dermal irritation and toxicity and no acute oral toxicity in rats^11^, the IC_50_ values for all Pro3–4 enhancers seem to be in an acceptable range. Furthermore, Pro2 did not cause any visible changes in rat skin after its topical application at 5% concentration^15^, i.e., 154 mM, indicating that the enhancer concentrations that reach viable cells are lower by several orders of magnitude than those applied to the skin. This assumption is supported by the viability of Pro3- and Pro4-treated skin (each applied at 1% in 60% PG for 24 or 48 h), which did not decline below 92% viability of untreated skin; this small decrease in viability was not significant compared to untreated skin. This effect may be caused by an unfavorable partitioning of a lipophilic enhancer from the lipophilic *stratum corneum* into a more hydrophilic viable epidermis. In addition, the levels of such ester enhancers are expected to be considerably reduced after their hydrolysis by esterases abundant in the *stratum corneum*. Such rapid ester hydrolysis was observed in several chemically related classes of enhancers^13,25,26^.

Concerning the morphological changes in cells after their incubation with Pro2–4 for 48 h, laser scanning confocal microscopy revealed a minor decrease in cell numbers at concentrations corresponding to the IC_15_ values with no detectable changes in cellular morphology (Fig. 3 and Supplementary Figure S1). At IC_85_ concentrations, the enhancers caused morphological changes typical of apoptotic cell death, such as retraction and rounding of the cells, formation of membrane blebs, redistribution of both F-actin and α-tubulin fibers, pyknosis, and karyorrhexis (Fig. 3). In addition, many cells detached from a substrate during the cell death process, indicating apoptosis rather than necrosis. Most of the remaining cells were morphologically unaffected and retained their typical morphological features. Importantly, no signs of necrosis that would initiate undesirable inflammatory reactions in vivo were detected. Those findings indicate apoptosis as a presumptive cell death pathway induced by the sublethal dose of all studied enhancers.

### Interaction of selected enhancers with human *stratum corneum*

**Figure 4:**
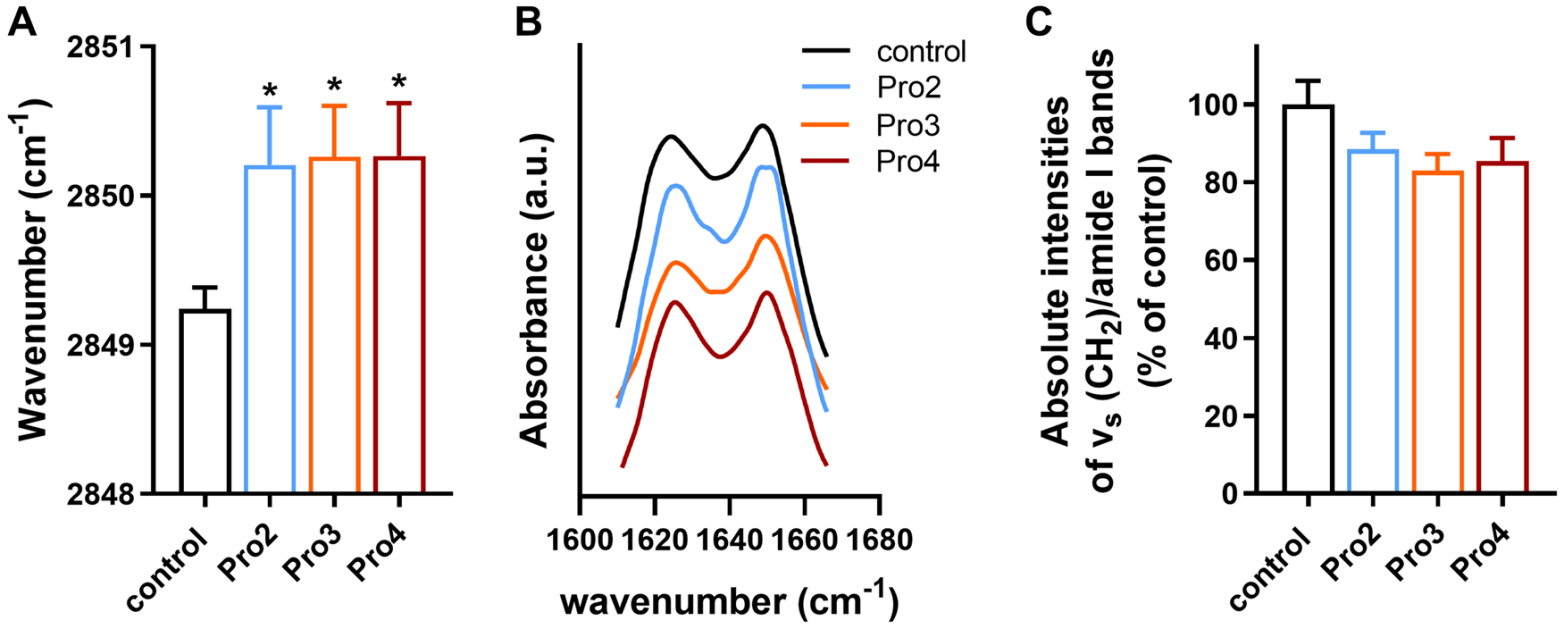
Interactions of the studied enhancers Pro2, Pro3, and Pro4 with human *stratum corneum* examined by infrared spectroscopy: methylene symmetric stretching wavenumbers indicative of decreased lipid chain order (**A**), amide I band with no significant effect on protein conformation (**B**), and relative intensities of the methylene symmetric stretching band to amide I band indicating no significant lipid extraction (**C**). Data are presented as the means ± SEM; n ≥ 3. *Statistically significant difference compared to control (60% PG without enhancer) at *p* < 0.05.

The interactions of Pro2–4 with isolated human *stratum corneum* were studied by Fourier-transform infrared spectroscopy (FTIR)^27^. The untreated and PG-treated stratum corneum samples had well-ordered lipid chains as indicated by the methylene symmetric stretching vibrations at around 2849 cm^−1^^28–30^. The Pro2–4-treated tissues showed polymethylene chains with numerous *gauche* defects^27,31^ as judged by an approximately 1 cm^−1^ shift to values over 2850 cm^−1^ (Fig. 4). Such changes are consistent with the compounds’ effects on the skin permeability and previous effects of similar amino acid-based enhancers^15,17,25^.

In contrast, structurally similar *N*-lauroyl sarcosine induced conformational changes in the *stratum corneum* proteins^32^; thus, we also probed the enhancer effects on the amide bands, coming from the peptide bonds with a minor contribution from ceramides. Treatment of the *stratum corneum* with 60% PG induced partial reorganization of the dominant α-helices into β-sheet conformation^27,33^, as suggested by splitting the amide I band at 1645 cm^−1^ into two bands with similar intensities at 1620 cm^−1^ and 1648 cm^−1^. Such a change is consistent with previous results^15,34,35^. Addition of the Pro2–4 enhancers into 60% PG changed neither the shape nor intensity of the amide bands, indicating that these enhancers do not modify the *stratum corneum* protein conformation. In addition, the ratios of lipid (methylene symmetric stretching) and protein (amide I) band intensities suggested that the enhancers did not extract the *stratum corneum* lipids.

Thus, Pro3 and Pro4 mostly interact with the *stratum corneum* lipids (similar to L-Pro2^15^). The observed changes in the infrared spectra of the enhancer-treated *stratum corneum* can be interpreted either as lipid fluidization or the creation of separated domains rich in enhancers. However, we cannot distinguish between these possibilities without deuterium-labeled compounds^36,37^. Nevertheless, either effect would plausibly explain the permeation-enhancing potency of these compounds.

### Reversibility of Pro2–4 effects on transepidermal water loss (TEWL) and electrical impedance

We further investigated the direct effects of the selected enhancers on the skin barrier assessed by TEWL and the electrical impedance of human skin in vitro. TEWL is a method commonly used in dermatology^38^ to study, for example, the irritant potential of chemicals^39^, skin barrier recovery^40^, or effect of topical formulations, and permeation enhancers^17,21,41–44^. Electrical impedance measures the opposition of the skin to alternating electrical current and is inversely proportional to the flux of ions. Thus, increased TEWL or decreased impedance indicates a barrier impairment^45,46^. Electrical impedance or conductance measurements have been used to screen new enhancers or assess the irritation potential of compounds^13,15,17,21,32,44,47–50^. Both TEWL and impedance can distinguish between reversible and irreversible effects of enhancers on skin permeability^17,21,40,44,51^.

**Figure 5:**
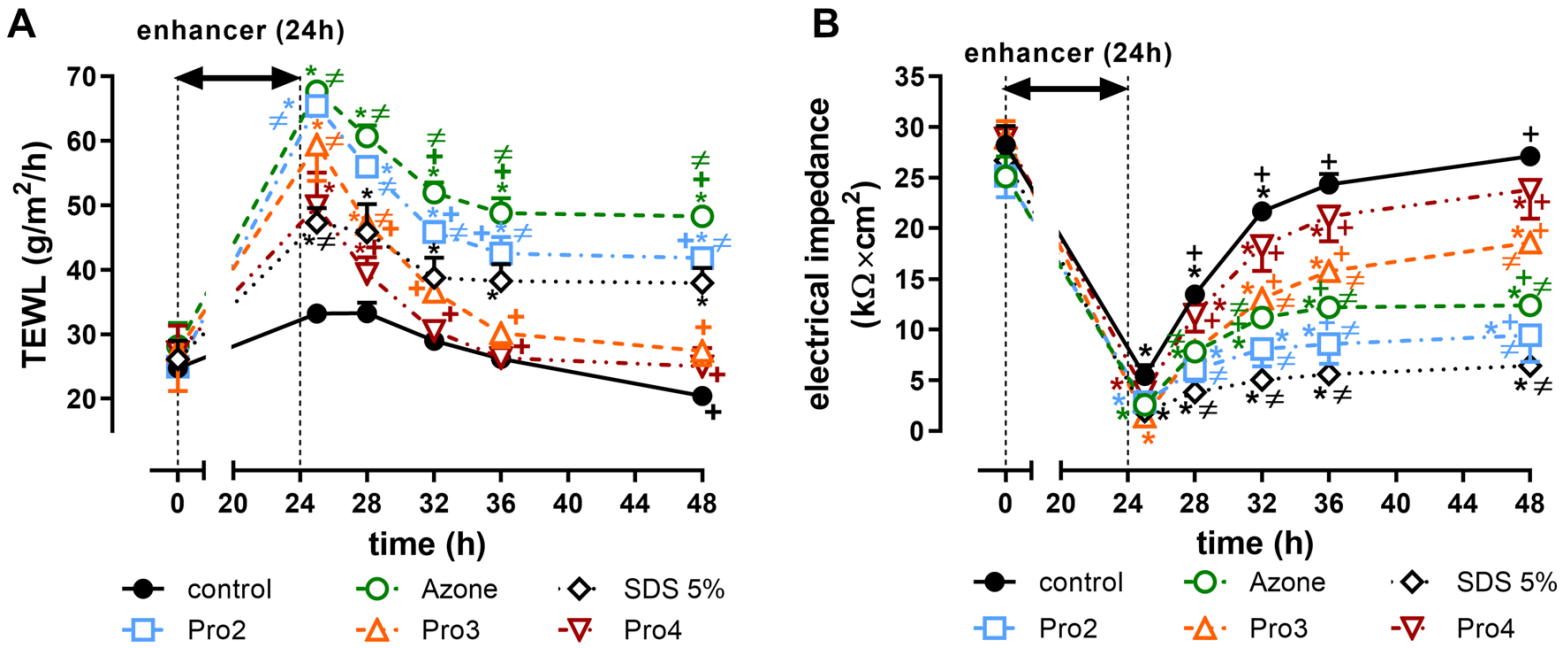
TEWL (**A**) and impedance (**B**) values before (0 h) and after 24-h application of 60% PG without (control) or with 1% enhancer (Pro2, Pro3, Pro4) or positive controls (1% Azone or 5% SDS); n = 4. *Statistically significant difference compared to the baseline value before the sample application at p < 0.05. ^+^Statistically significant compared to the value after the sample removal (25 h) at p < 0.05. ^≠^Statistically significant compared to the control (60% PG without enhancer) at the same time point, at p < 0.05. The values are given in Supplementary Table S1.

Treatment of human skin with Pro2, Pro3, and Pro4 in vitro for 24 h increased the TEWL values approximately two-fold (50–65 g/m^2^/h) over the baseline values before treatment (around 27 g/m^2^/h). Control 60% PG solution without enhancers did not increase TEWL significantly (Fig. 5, Supplementary Table S1). These results are consistent with the permeation enhancement potency of these compounds—such a correlation has been observed previously^45^. After Pro3 and Pro4 removal from the skin, TEWL declined to values similar to baseline TEWL within 8 h, indicating fast and full reversibility (Fig. 5). In contrast, Pro2-induced TEWL increase was only partly reversible within 24 h.

The impedance of the skin treated with Pro2, Pro3, Pro4, and control (60% PG without enhancers) for 24 h was significantly lower (5–21 times), respectively, compared to baseline impedance (25–29 kΩ × cm^2^) (Fig. 5). The impedance of Pro4 and control-treated skin recovered to values similar to baseline within 24 h, whereas the Pro3 and Pro2-treated skin recovered incompletely. Thus, the longer the acyl chain, the greater recovery was observed. As TEWL and impedance are methods sensitive to irritant skin reactions^52^, Pro3 and, in particular Pro4, can be expected to have low irritation potential. This assumption is consistent with the lack of TEWL and impedance reversibility in 1% Azone (*N-*dodecylazepan-2-one) and 5% sodium dodecyl sulfate (SDS)^53–55^-treated skin.

### Conclusion

In conclusion, we prepared three series of new skin permeation enhancers based on proline, 4-hydroxyproline, and pyrrolidone carboxylic acid. Permeation studies identified dodecyl prolinates with *N-*acetyl, propionyl, and butyryl chains Pro2, Pro3, and Pro4 as potent enhancers for model drugs theophylline and diclofenac. These enhancers had acceptable in vitro cellular toxicities and preferentially interacted with the skin barrier lipids with negligible effects on the *stratum corneum* protein structures. The impacts of Pro3 and, in particular, Pro4 on an in vitro transepidermal water loss and electrical impedance of human skin were fully reversible, which is advantageous for their potential in vivo use as the skin barrier would quickly recover after the drug/enhancer administration.

## Materials and methods

All methods were performed in accordance with the relevant guidelines and regulations.

### Synthesis

The product synthesis, characterization, and yields are given in the Supplementary data.

### Skin

Human skin was obtained from Caucasian patients who underwent abdominal surgery, with their written informed consent. The study was approved by the ethics committee of the Sanus surgical center (4/6/2015). Subcutaneous fat was carefully removed from the tissue, and the remaining full-thickness skin fragments were washed with water and saline, gently blotted dry, and stored at − 20 °C.

### Donor samples

The donor samples for permeation experiments contained a model drug (5% TH, or 3% DC) in 60% aqueous propylene glycol (PG) with or without the studied compound at 1% (w/w). Donor samples for reversibility and infrared studies contained 1% enhancer in 60% PG. All samples were thoroughly mixed, incubated at 32 °C for 24 h, and, if needed, carefully resuspended before applying on the skin. The effects of the studied enhancers on the solubility of model drugs were assessed as described previously^17^.

### Permeation experiments

The permeation-enhancing potencies of the prepared compounds were evaluated on human skin in Franz diffusion cells. Human skin was slowly thawed, cut into 2 × 2 cm pieces, fixed in Teflon holders with 1-cm^2^ circular permeation areas, and mounted into Franz cells epidermal side up. The acceptor compartment (15.9 ± 0.1 ml) was filled with phosphate-buffered saline at pH 7.4 with 0.005% gentamicin (PBS) and stirred at 32 °C throughout the experiment. The skin integrity was checked by electrical impedance (any skin sample with the impedance < 10 kΩ was excluded). Then, 100 µl of a donor sample (infinite dose) was applied to the skin and covered with a glass slide. Acceptor phase samples (300 µl) were collected at predetermined time intervals for 48 h, to reach steady-state and were replaced by the same volume of fresh PBS. The sink conditions were maintained (the PBS solubilities of TH and DC were 7.65 ± 0.02 mg/ml and 9.45 ± 0.15 mg/ml, respectively). The drug concentrations were measured by HPLC (see supplementary data).

The cumulative amount of TH and DC permeated through the skin was corrected for the acceptor phase replacement and plotted against time. The flux of the model drug through the skin *J*_*ss*_ (µg/cm^2^/h) was calculated from the linear region of this plot. The enhancement ratio (ER) was calculated as the ratio of flux with and without an enhancer.

After the permeation experiment, the cells were dismounted, the skin was carefully washed with PBS, and the permeation area of the skin (1 cm^2^) was punched off. After heating the skin fragments to 80 °C for 1 min, the epidermis (E) was peeled off the dermis (D), individually weighed and extracted by 1 and 2 ml, respectively, of an extraction solvent (identical to the mobile phase for the drug HPLC analysis, see supplementary data) for 24 h. The drug concentration was measured by HPLC (see supplementary data).

### Reversibility of enhancer effects on transepidermal water loss (TEWL) and electrical impedance

Human skin was mounted in Franz diffusion cells in the same way as for the permeation studies. After 1-h equilibration, basal TEWL and impedance values were recorded. Then, 1% enhancer in 60% PG, 60% PG as a negative control, 1% Azone in 60% PG, or 5% SDS in 60% PG as positive (irreversible) controls was applied on the skin (100 µl/cm^2^) for 24 h. The residual sample was washed with PBS and gently blotted dry. At 1, 4, 8, 12, and 24 h after sample removal, TEWL, and impedance values were recorded. TEWL was measured using AquaFlux AF 200 (Biox Systems Ltd, London, UK), employing the condenser-chamber measurement method at 26 ± 1.0 °C and 43 ± 3% relative air humidity. Electrical impedance was measured by LCR meter 4080 (Conrad electronic, Hirschau, Germany, measuring range 20 Ω–10 MΩ, error at kΩ values < 0.5%) operated in parallel mode with an alternating frequency of 120 Hz, which was also used in our previous work^21,44^.

### Fourier-transform infrared (FTIR) spectroscopy

The effects of selected enhancers on the organization of skin barrier lipids and proteins were investigated by FTIR spectroscopy. The epidermis was obtained from human skin by heat separation, and the *stratum corneum* was isolated using trypsin as described before^56^. The donor solution (60% PG with or without 1% enhancer; 10 µl per mg of hydrated *stratum corneum*) was applied to the *stratum corneum*. After 24-h incubation at 32 °C, the donor samples were gently removed from the *stratum corneum* by a cotton swab, and FTIR spectra were recorded on a Nicolet 6700 FT FTIR spectrometer (Thermo Scientific, Waltham, USA) equipped with a single-reflection MIRacle attenuated total reflectance ZnSe crystal. The spectra were generated by co-addition of 128 scans recorded at a 2-cm^−1^ resolution.

### Cellular toxicity

The toxicities of selected enhancers are presented as their IC_50_ (concentration of enhancer, which leads to 50% decrease in the cell viability) in vitro on two different cell lines, 3T3 mouse fibroblasts (American Type Culture Collection, ATCC, Manassas, USA) and HaCaT spontaneously immortalized human keratinocytes (Cell Lines Service, Eppelheim, Germany). Cell viability was determined via 3-(4,5-dimethylthiazol-2-yl)-2,5-diphenyltetrazolium bromide (MTT) and neutral red (NR) uptake assays after 48-h incubation with the enhancers (see Supplementary data for details).

### Laser scanning confocal microscopy

Morphological changes of the cells (3T3 and HaCaT) were evaluated via laser scanning confocal microscopy. After 48-h incubation of cells in the same way as in the toxicity studies, using the enhancer concentration corresponding to their IC_15_ and IC_85_, cell samples were prepared for confocal microscopy (see Supplementary data for details). Finally, eight focal planes (pinhole diameter = 19.16 μm) were taken of each sample using a Nikon A1 + confocal system (Nikon, Tokyo, Japan) equipped with NIS Elements AR 4.20 software (Laboratory Imaging, Prague, Czech Republic).

### Skin viability after topical enhancer application

Freshly excised full thickness human skin was incubated in Dulbecco’s modified Eagle’s medium (DMEM, Lonza, Verviers, Belgium) supplemented with 10% heat-inactivated fetal bovine serum (Merck, Darmstadt, Germany), and 1% penicillin/streptomycin + 1% amphotericin B solution (Lonza, Verviers, Belgium) at 37 °C in a humidified atmosphere of 5% CO2. Skin fragments were treated with 1% enhancer in 60% PG, 20 µl/cm^2^, 60% PG alone or left untreated for 24 or 48 h. Then the skin was washed rinsed with distilled water and gently blotted dry. Skin viability was evaluated using the 2,3,5-triphenyltetrazolium chloride (TTC) reduction test^57^. Punch biopsies of 5 mm diameter were incubated in 1 ml of 1.5% TTC and 3% sodium succinate in phosphate buffered saline at pH 7.4 at 37 °C under nitrogen in the dark. After 1 h, the skin samples were dried and the dark red formazan derivative produced by the mitochondrial enzymes was extracted with 1 ml of 2-methoxyethanol for 12 h. Absorbance was measured at 490 nm using Tecan Infinite 200 M plate reader (Tecan, Grödig, Austria) and corrected using skin samples devitalized by boiling as a negative control. The viability of skin exposed to the different preparations was expressed as a percentage of untreated control skin.

### Data treatment

Obtained data were analyzed with GraphPad Prism software (v. 9.3.1, GraphPad Software, San Diego, USA). One-way ANOVA with Bonferroni´s multiple comparisons post hoc test or two-way ANOVA with Dunnett´s multiple comparison test was used. The differences were considered significant at p < 0.05. Toxicity is represented as the concentration of compound inducing a 50% decrease of the cell viability (IC_50_), which was calculated using GraphPad. Data are presented as means ± SEM, and the number of replicates (*n*) is given in the appropriate figures.

## Supplementary Information

Supplementary Information.

## Data Availability

The datasets used and/or analysed during the current study available from the corresponding author on reasonable request.

## Abbreviations

DC: Diclofenac
ER: Enhancement ratio
Hyp: Hydroxyproline
MTT: 3-(4,5-Dimethylthiazol-2-yl)-2,5-diphenyltetrazolium bromide
NR: Neutral red dye, 3-amino-7-dimethylamino-2-methylphenazine hydrochloride
PBS: Phosphate-buffered saline
PCA: Pyrrolidone carboxylic acid
PG: Propylene glycol
Pro: Proline
SDS: Sodium dodecyl sulfate
TEWL: Transepidermal water loss
TH: Theophylline
