# Supplementary material for: Proline, hydroxyproline, and pyrrolidone carboxylic acid derivatives as highly efficient but reversible transdermal permeation enhancers
Source: Sci Rep. 2022 Nov 14;12:19495. doi: 10.1038/s41598-022-24108-6 (PMC9663686; doi:10.1038/s41598-022-24108-6)
Supplement: Supplementary file 1 — Supplementary Information. [file 41598_2022_24108_MOESM1_ESM.docx]

**SUPPLEMENTARY DATA**

**Proline, hydroxyproline, and pyrrolidone carboxylic acid derivatives as highly efficient but reversible transdermal permeation enhancers**

Monika Kopečná^1^, Miloslav Macháček^2^, Jaroslav Roh^3^, Kateřina Vávrová^1,*^

*^1^Skin Barrier Research Group, Charles University, Faculty of Pharmacy in Hradec Králové, Akademika Heyrovského 1203, 50005 Hradec Králové, Czech Republic*

^2^*Department of Biochemical Sciences, Charles University, Faculty of Pharmacy in Hradec Králové, Akademika Heyrovského 1203, 50005 Hradec Králové, Czech Republic*

^3^*Department of Organic and Bioorganic Chemistry, Charles University, Faculty of Pharmacy in Hradec Králové, Akademika Heyrovského 1203, 50005 Hradec Králové, Czech Republic*

* [katerina.vavrova@faf.cuni.cz](mailto:katerina.vavrova@faf.cuni.cz)

Contents

[Supplementary Methods 1](#_Toc103341442)

[Synthesis 1](#_Toc103341443)

[Characterization of prepared compounds 2](#_Toc103341444)

[High-performance liquid chromatography (HPLC) 7](#_Toc103341445)

[Cellular toxicity assessment 7](#_Toc103341446)

[Laser scanning confocal microscopy 8](#_Toc103341447)

[Figure S1 9](#_Toc103341448)

[Table S1 10](#_Toc103341449)

[References 10](#_Toc103341450)

## Supplementary Methods

### Synthesis

Chemicals for the synthesis and purification of studied compounds were purchased from Merck (Darmstadt, Germany). Reactions were monitored on aluminum TLC plates with silica gel 60 F254, and products were purified using column chromatography on Merck Kieselgel 60 (0.040‑0.063 mm). The identity and structure of prepared compounds were confirmed by ^1^H-NMR and ^13^C-NMR spectroscopy (Varian Mercury Vx BB 300 or VNMR S500 NMR spectrometer; Varian, Palo Alto, CA, USA; chemical shifts were indirectly referred to tetramethylsilane (TMS) *via* the solvent signal and reported as δ values in parts per million (ppm)), mass spectrometry (Agilent 500 Ion Trap LC/MS; Agilent Technologies, Santa Clara, CA, USA) and infrared spectroscopy (Nicolet 6700 IR spectrometer equipped with an ATR crystal; Thermo Scientific, Waltham, USA).

*Synthesis of proline derivatives Pro2 – Pro8 and hydroxyproline derivatives Hyp2 – Hyp5.* All amino acids were used as racemates. Dodecyl prolinate hydrochloride (Pro dodecyl ester) and dodecyl 4-hydroxyprolinate hydrochloride (Hyp dodecyl ester) were prepared by heating racemic proline or racemic 4-hydroxyproline with dodecanol (5 equiv.) under argon at 120°C and 70°C, respectively. Dry hydrogen chloride was bubbled through this mixture for 15 min every 2 h.^1^ After 7 h, the reaction mixture was allowed to cool down to room temperature (rt) and stirred overnight. Pro dodecyl ester hydrochloride was purified by column chromatography first with hexane/ethyl acetate 1:1 to remove dodecanol, then ethyl acetate, and finally methanol. Hyp dodecyl ester hydrochloride was precipitated from the reaction mixture with acetone. Both products were recrystallized from diethyl ether.

Next, Pro dodecyl ester hydrochloride or Hyp dodecyl ester hydrochloride was dissolved in dry chloroform with *N,N’*-dicyclohexylcarbodiimide (DCC; 1  equiv.) and 4-dimethylaminopyridine (DMAP; 1 equiv.) under argon. At 0°C, an appropriate carboxylic acid (0.9 equiv.) was added dropwise and stirred at rt overnight. The reaction mixture was evaporated and dissolved in ethyl acetate, and the precipitated *N, N’*‑dicyclohexylurea was filtered off. The filtrate was extracted with saturated NaHCO_3_, dried over Na_2_SO_4,_ and purified by column chromatography on silica gel using hexane/ethyl acetate 4:1 and 2:1. The product characterization and yields are given below.

*Synthesis of 5-oxoproline derivatives PCA2 – PCA5.* Dodecyl 5-oxoprolinate (PCA dodecyl ester) was prepared by dissolving PCA with dodecanol (1.1 equiv.) and DMAP (0.1 equiv.) in dry dichloromethane under argon. At 0 °C, DCC (1.1 equiv.) was added dropwise, and the reaction mixture was stirred at rt overnight. The reaction mixture was evaporated and dissolved in ethyl acetate, and the precipitated *N, N’*‑dicyclohexylurea was filtered off. The filtrate was extracted with 0.1 M HCl and saturated NaHCO_3_, dried over Na_2_SO_4,_ and purified by column chromatography on silica gel using hexane/ethyl acetate 2:1 and 1:1.

To synthesize PCA3 – PCA5, PCA dodecyl ester and diisopropylethylamine (DIPEA, 3 equiv.) were dissolved in dry toluene under argon, and an appropriate acyl chloride (2 equiv.) was added dropwise, and the reaction mixture was stirred at rt overnight. The reaction mixture was evaporated, the residue dissolved in chloroform and extracted by 0.1 M HCl, saturated NaHCO_3_ and water, and dried over Na_2_SO_4_. The products were obtained by column chromatography on silica gel using hexane/ethyl acetate 9:1.

For PCA2, PCA dodecyl ester was mixed with DMAP (2 equiv.) in dry chloroform under argon, acetanhydride (1 equiv.) was added and stirred for 5 h. The reaction mixture was then extracted by saturated NaCl and dried over Na_2_SO_4_. PCA2 was obtained by column chromatography on silica gel using hexane/ethyl acetate 9:1. The product characterization and yields are given below.

### Characterization of prepared compounds


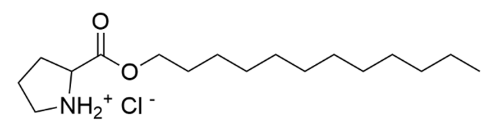


2-((dodecyloxy)carbonyl)pyrrolidin-1-ium chloride (**Pro dodecyl ester hydrochloride**): Yield 21 % as a white waxy crystals; T_m_ = 58-62 °C; M_w_ = 319.91. ^1^H NMR (300 MHz, DMSO-d6) δ 9.54 (broad s, 1H), 4.32 – 4.25 (m, 1H), 4.20 – 4.05 (m, 2H), 3.25 – 3.08 (m, 2H), 2.29 – 2.17 (m, 1H), 2.01 – 1.79 (m, 3H), 1.65 – 1.53 (m, 2H), 1.37 – 1.14 (m, 18H), 0.91 – 0.79 (m, 3H). ^13^C NMR (75 MHz, DMSO) δ 169.34, 65.91, 58.61, 45.34, 31.50, 29.24, 29.22, 29.18, 29.12, 28.92, 28.81, 28.12, 28.09, 25.39, 23.32, 22.30, 14.14. IR (ATR): ν_max_ 2920; 2850; 1730; 1473; 1245; 1168; 1049 cm^-1^. MS (APCI, pos): m/z 284.5 [M+H]^+^.


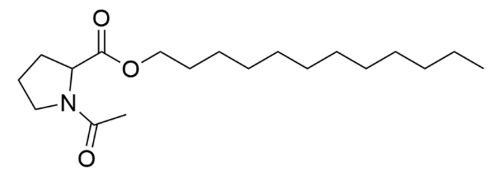


Dodecyl ester of *N*-acetyl proline (**Pro2**): Yield 90 % as a colourless oil; M_w_ = 325.49. ^1^H NMR (500 MHz, CDCl_3_) δ 4.48 (dd, *J* = 8.7, 3.7 Hz, 0.75H), 4.36 (dd, *J* = 8.6, 2.7 Hz, 0.25H), 4.11 (t, *J* = 6.8 Hz, 2H), 3.69 – 3.61 (m, 1H), 3.54 – 3.47 (m, 1H), 2.23 – 2.14 (m, 1H), 2.10 – 1.90 (m, 6H), 1.67 – 1.59 (m, 2H), 1.37 – 1.22 (m, 18H), 0.88 (t, *J* = 6.9 Hz, 3H). ^13^C NMR (126 MHz, CDCl_3_) δ 172.39, 172.20, 169.46, 169.27, 65.67, 65.17, 60.27, 58.59, 47.68, 46.24, 31.86, 31.48, 29.59, 29.58, 29.52, 29.49, 29.47, 29.42, 29.29, 29.17, 29.10, 28.51, 25.78, 25.75, 24.70, 22.77, 22.64, 22.21, 22.19, 14.06. IR (ATR): ν_max_ 2920; 2851; 1740; 1629; 1470; 1417; 1175 cm^-1^. MS (ESI, pos): m/z 348.3 [M+Na]^+^.


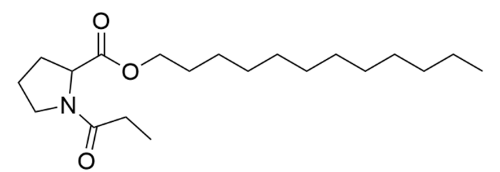


Dodecyl ester of *N*-propionyl proline (**Pro3**): Yield 95 % as a colourless oil; M_w_ = 339.52. ^1^H NMR (300 MHz, CDCl_3_) δ 4.47 (dd, *J* = 8.6, 3.6 Hz, 0.75H), 4.36 (dd, *J* = 8.5, 2.7 Hz, 0.25H), 4.15 – 4.04 (m, 2H), 3.67 – 3.55 (m, 1H), 3.53 – 3.42 (m, 1H), 2.43 – 1.83 (m, 8H), 1.69 – 1.54 (m, 2H), 1.36 – 1.22 (m, 16H), 1.14 (t, *J* = 7.5 Hz, 3H), 0.94 – 0.79 (m, 3H). ^13^C NMR (75 MHz, CDCl_3_) δ 172.57, 172.53, 65.63, 65.15, 59.47, 58.71, 46.80, 46.36, 31.87, 31.53, 29.61, 29.59, 29.53, 29.51, 29.48, 29.45, 29.31, 29.24, 29.19, 29.12, 28.52, 27.58, 27.45, 25.79, 25.77, 24.72, 22.65, 22.55, 14.09, 8.70. IR (ATR): ν_max_ 2924; 2854; 2360; 1744; 1655; 1422; 1184 cm^-1^. MS (APCI, pos): m/z 340.5 [M+H]^+^.


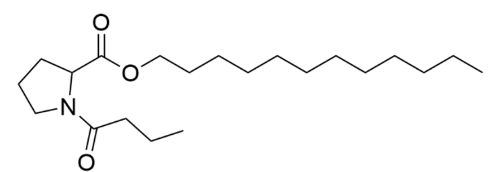


Dodecyl ester of *N*-butyryl proline (**Pro4**): Yield 90 % as a colourless oil; M_w_ = 353.55. ^1^H NMR (300 MHz, CDCl_3_) δ 4.47 (dd, *J* = 8.4, 3.7 Hz, 0.75H), 4.37 (dd, *J* = 8.5, 2.7 Hz, 0.25H), 4.15 – 4.04 (m, 2H), 3.68 – 3.57 (m, 1H), 3.60 – 3.42 (m, 1H), 2.37 – 1.85 (m, 6H), 1.75 – 1.56 (m, 4H), 1.36 – 1.20 (m, 18H), 0.96 (t, *J* = 7.4 Hz, 3H), 0.90 – 0.82 (m, 3H). ^13^C NMR (75 MHz, CDCl_3_) δ 172.53, 172.45, 171.97, 171.87, 65.63, 65.14, 59.54, 58.67, 46.96, 46.25, 36.34, 36.26, 31.88, 31.52, 29.61, 29.59, 29.54, 29.48, 29.31, 29.25, 29.20, 28.52, 25.80, 24.74, 22.65, 22.56, 18.29, 18.09, 14.09, 13.87. IR (ATR): ν_max_ 2924; 2854; 2360; 1744; 1653; 1421; 1184 cm^-1^. MS (APCI, pos): m/z 354.5 [M+H]^+^.


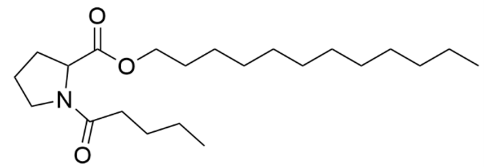


Dodecyl ester of *N*-pentanoyl proline (**Pro5**): Yield 91 % as a colourless oil; M_w_ = 367.57. ^1^H NMR (300 MHz, CDCl_3_) δ 4.47 (dd, *J* = 8.5, 3.6 Hz, 0.75H), 4.37 (dd, *J* = 8.5, 2.7 Hz, 0.25H), 4.15 – 4.04 (m, 2H), 3.68 – 3.56 (m, 1H), 3.60 – 3.42 (m, 1H), 2.39 – 1.81 (m, 6H), 1.69 – 1.54 (m, 4H), 1.40 – 1.19 (m, 20H), 0.95 – 0.82 (m, 6H). ^13^C NMR (75 MHz, CDCl_3_) δ 172.54, 172.00, 65.14, 58.67, 46.96, 34.16, 31.88, 31.52, 29.61, 29.59, 29.54, 29.49, 29.31, 29.25, 29.21, 28.52, 26.96, 26.73, 25.80, 24.75, 22.65, 22.46, 14.09, 13.87. IR (ATR): ν_max_ 2925; 2854; 2362; 1744; 1654; 1420; 1184 cm^-1^. MS (APCI, pos): m/z 368.5 [M+H]^+^.


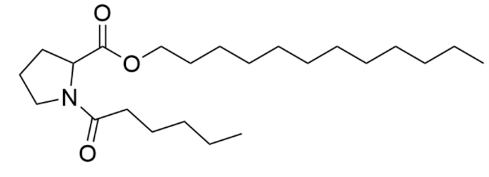


Dodecyl ester of *N*-hexanoyl proline (**Pro6**): Yield 84 % as a colourless oil; M_w_ = 381.60. ^1^H NMR (300 MHz, CDCl_3_) δ 4.47 (dd, *J* = 8.6, 3.6 Hz,0.9H), 4.37 (dd, *J* = 8.5, 2.7 Hz, 0.1H), 4.15 – 4.04 (m, 2H), 3.67 – 3.54 (m, 1H), 3.55 – 3.44 (m, 1H), 2.35 – 1.85 (m, 6H), 1.70 – 1.55 (m, 4H), 1.33 – 1.22 (m, 22H), 0.92 – 0.83 (m, 6H). ^13^C NMR (75 MHz, CDCl_3_) δ 172.53, 172.03, 65.63, 65.14, 59.55, 58.68, 46.96, 46.28, 34.43, 34.34, 31.88, 31.58, 31.55, 31.52, 29.62, 29.60, 29.55, 29.49, 29.32, 29.25, 29.21, 28.53, 25.81, 24.75, 24.32, 22.66, 22.48, 14.09, 13.94. IR (ATR): ν_max_ 2924; 2854; 1744; 1654; 1419; 1182 cm^-1^. MS (APCI, pos): m/z 382.5 [M+H]^+^.


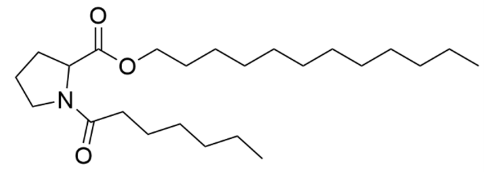


Dodecyl ester of *N*-heptanoyl proline (**Pro7**): Yield 89 % as a colourless oil; M_w_ = 395.63. ^1^H NMR (300 MHz, CDCl_3_) δ 4.47 (dd, *J* = 8.5, 3.7 Hz, 0.8H), 4.37 (dd, *J* = 8.5, 2.7 Hz, 0.2H), 4.15 – 4.04 (m, 2H), 3.68 – 3.58 (m, 1H), 3.54 – 3.43 (m, 1H), 2.35 – 1.85 (m, 6H), 1.69 – 1.54 (m, 4H), 1.34 – 1.21 (m, 24H), 0.91 – 0.82 (m, 6H). ^13^C NMR (75 MHz, CDCl_3_) δ 172.54, 172.47, 172.12, 172.00, 65.62, 65.13, 59.54, 58.67, 46.95, 46.27, 34.49, 34.39, 31.88, 31.62, 31.61, 31.52, 29.61, 29.59, 29.54, 29.53, 29.49, 29.47, 29.31, 29.25, 29.21, 29.15, 29.08, 29.06, 28.53, 25.80, 24.85, 24.75, 24.58, 22.65, 22.57, 22.50, 14.08, 14.03. IR (ATR): ν_max_ 2925; 2854; 1745; 1654; 1420; 1182 cm^-1^. MS (APCI, pos): m/z 396.6 [M+H]^+^.


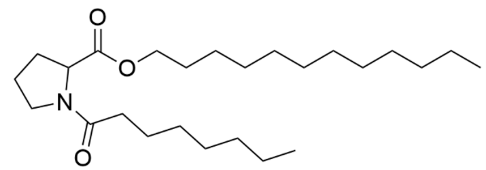


Dodecyl ester of *N*-octanoyl proline (**Pro8**): Yield 92 % as a colourless oil; M_w_ = 409.66. ^1^H NMR (500 MHz, CDCl_3_) δ 4.47 (dd, *J* = 8.7, 3.8 Hz, 0.75H), 4.37 (dd, *J* = 8.6, 2.6 Hz, 0.25H), 4.15 – 4.06 (m, 2H), 3.66 – 3.58 (m, 1H), 3.54 – 3.45 (m, 1H), 2.36 – 1.86 (m, 6H), 1.69 – 1.56 (m, 4H), 1.36 – 1.21 (m, 26H), 0.90 – 0.84 (m, 6H). ^13^C NMR (126 MHz, CDCl_3_) δ 172.53, 172.01, 65.62, 65.13, 59.56, 58.69, 46.96, 46.27, 34.49, 34.40, 31.88, 31.69, 31.52, 29.62, 29.60, 29.59, 29.55, 29.53, 29.49, 29.47, 29.38, 29.35, 29.31, 29.25, 29.21, 29.16, 29.10, 29.08, 28.56, 28.54, 25.81, 24.90, 24.75, 24.64, 22.65, 22.60, 22.57, 14.08, 14.04. IR (ATR): ν_max_ 2924; 2854; 1744; 1655; 1420; 1180 cm^-1^. MS (APCI, pos): m/z 410.5 [M+H]^+^.


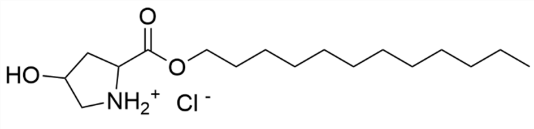


2-((dodecyloxy)carbonyl)-4-hydroxypyrrolidin-1-ium chloride (**Hyp dodecyl ester hydrochloride**): Yield 22 % as a white waxy crystals; T_m_ = 116-121 °C; M_w_ = 335.91. ^1^H NMR (300 MHz, CDCl_3_) δ 9.43 (broad s, 2H), 5.15 (broad s, 1H), 4.81 – 4.64 (m, 2H), 4.27 – 4.09 (m, 2H), 3.76 (d, *J* = 12.4 Hz, 1H), 3.61 (dd, *J* = 12.5, 3.6 Hz, 1H), 2.55 (dd, *J* = 13.6, 7.8 Hz, 1H), 2.25 – 2.09 (m, 1H), 1.72 – 1.59 (m, 2H), 1.34 – 1.20 (m, 18H), 0.87 (t, *J* = 6.6 Hz, 3H). ^13^C NMR (75 MHz, CDCl_3_) δ 169.37, 69.74, 67.11, 58.32, 54.65, 37.70, 31.89, 29.65, 29.62, 29.60, 29.52, 29.34, 29.24, 28.34, 25.73, 22.66, 14.09. IR (ATR): ν_max_ 2921; 2850; 1746; 1594; 1276; 1237; 1182; 1104 cm^-1^. MS (APCI, pos): m/z 300.5 [M+H]^+^.


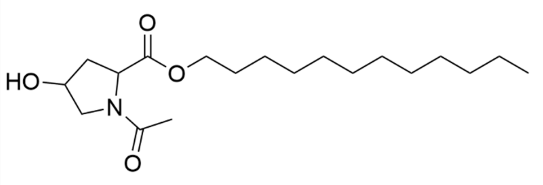


Dodecyl 1-acetyl-4-hydroxypyrrolidine-2-carboxylate (**Hyp2**): Yield 88 % as a colourless oil; M_w_ = 341.49. ^1^H NMR (500 MHz, CDCl_3_) δ 4.61 – 4.48 (m, 2H), 4.12 (t, *J* = 6.8 Hz, 2H), 3.86 – 3.76 (m, 1H), 3.59 – 3.48 (m, 1H), 2.49 – 2.41 (m, 0.4H), 2.32 – 2.25 (m, 0.6H), 2.24 – 2.18 (m, 0.4H), 2.14 – 2.10 (m, 0.6H), 2.08 (s, 2H, COCH_3_), 1.98 (s, 1H, COCH_3_), 1.68 – 1.60 (m, 2H), 1.38 – 1.21 (m, 18H), 0.88 (t, *J* = 6.9 Hz, 3H). ^13^C NMR (126 MHz, CDCl_3_) δ 172.38, 172.26, 170.37, 169.67, 70.31, 68.69, 65.88, 65.38, 58.87, 57.53, 55.78, 54.49, 39.77, 38.05, 31.89, 31.88, 29.67, 29.63, 29.61, 29.59, 29.56, 29.52, 29.50, 29.45, 29.32, 29.31, 29.21, 29.13, 28.49, 25.78, 25.75, 22.66, 22.22, 21.63, 14.09. IR (ATR): ν_max_ 2919; 2850; 1738; 1633; 1462; 1417; 1190 cm^-1^. MS (APCI, pos): m/z 342.3 [M+H]^+^.


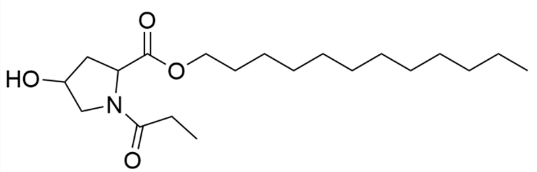


Dodecyl 4-hydroxy-1-propionylpyrrolidine-2-carboxylate (**Hyp3**): Yield 68 % as a colourless oil; M_w_ = 355.52. ^1^H NMR (500 MHz, CDCl_3_) δ 4.59 – 4.46 (m, 2H), 4.19 – 4.06 (m, 2H), 3.86 – 3.80 (m, 0.25H), 3.75 (dd, *J* = 10.8, 4.6 Hz, 0.75H), 3.55 (dd, *J* = 12.6, 4.5 Hz, 0.25H), 3.53 – 3.47 (m, 0.75H), 2.34 – 2.03 (m, 4H), 1.67 – 1.57 (m, 2H), 1.35 – 1.20 (m, 18H), 1.16 – 1.09 (m, 3H), 0.87 (t, *J* = 6.8 Hz, 3H). ^13^C NMR (126 MHz, CDCl_3_) δ 172.97, 172.51, 70.32, 68.54, 65.83, 65.34, 58.20, 57.64, 54.93, 54.56, 39.71, 37.82, 31.88, 29.62, 29.60, 29.59, 29.55, 29.50, 29.31, 29.22, 28.50, 27.65, 25.79, 22.65, 14.08, 8.68. IR (ATR): ν_max_ 2922; 2852; 1747; 1612; 1467; 1190 cm^-1^. MS (APCI, pos): m/z 356.5 [M+H]^+^.


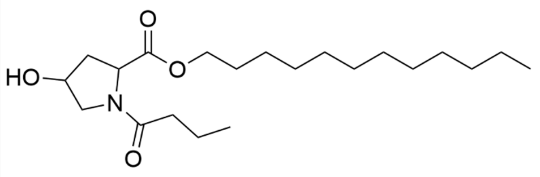


Dodecyl 1-butyryl-4-hydroxypyrrolidine-2-carboxylate (**Hyp4**): Yield 68 % as a colourless oil; M_w_ = 369.55. ^1^H NMR (500 MHz, CDCl_3_) δ 4.60 – 4.46 (m, 2H), 4.16 – 4.05 (m, 2H), 3.82 (d, *J* = 12.5 Hz, 0.2H), 3.76 (dd, *J* = 10.8, 4.6 Hz, 0.8H), 3.56 (dd, *J* = 12.6, 4.6 Hz, 0.2H), 3.51 (d, *J* = 10.4 Hz, 0.8H), 2.32 – 2.22 (m, 3H), 2.12 – 2.03 (m, 1H), 1.71 – 1.58 (m, 4H), 1.36 – 1.20 (m, 18H), 0.99 – 0.90 (m, 3H), 0.87 (t, *J* = 6.8 Hz, 3H). ^13^C NMR (126 MHz, CDCl_3_) δ 172.47, 172.32, 70.35, 68.57, 65.83, 65.34, 58.27, 57.61, 55.10, 54.46, 39.69, 37.84, 36.38, 35.61, 31.88, 29.63, 29.60, 29.59, 29.56, 29.53, 29.50, 29.46, 29.32, 29.31, 29.23, 29.15, 28.51, 25.80, 25.78, 22.66, 18.25, 18.11, 14.08, 13.84, 13.81. IR (ATR): ν_max_ 2921; 2852; 1743; 1619; 1463; 1184 cm^-1^. MS (APCI, pos): m/z 370.8 [M+H]^+^.


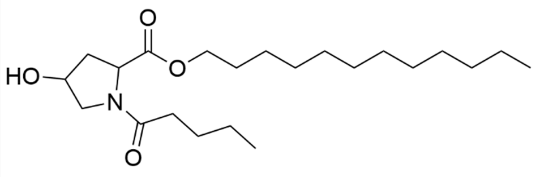


Dodecyl 4-hydroxy-1-pentanoylpyrrolidine-2-carboxylate (**Hyp5**): Yield 68 % as a colourless oil; M_w_ = 383.57. ^1^H NMR (500 MHz, CDCl_3_) δ 4.59 – 4.46 (m, 2H), 4.16 – 4.05 (m, 2H), 3.85 – 3.78 (m, 0.2H), 3.76 (dd, *J* = 10.8, 4.6 Hz, 0.8H), 3.55 (dd, *J* = 12.6, 4.5 Hz, 0.2H), 3.54 – 3.48 (m, 0.8H), 2.32 – 2.25 (m, 2H), 2.27 – 2.14 (m, 1H), 2.12 – 2.03 (m, 1H), 1.66 – 1.56 (m, 4H), 1.40 – 1.19 (m, 20H), 0.93 – 0.89 (m, 3H), 0.87 (t, *J* = 6.9 Hz, 3H). ^13^C NMR (126 MHz, CDCl_3_) δ 172.50, 172.42, 70.33, 68.56, 65.82, 65.33, 58.26, 57.59, 55.07, 37.82, 34.22, 33.44, 31.88, 29.62, 29.60, 29.56, 29.50, 29.46, 29.32, 29.23, 28.50, 26.87, 26.71, 25.79, 22.65, 22.39, 14.08, 13.83. IR (ATR): ν_max_ 2924; 2854; 1744; 1625; 1454; 1185 cm^-1^. MS (APCI, pos): m/z 384.7 [M+H]^+^.


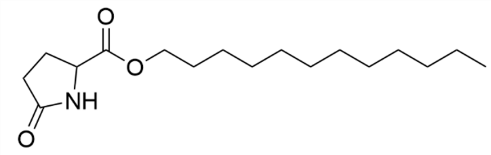


Dodecyl 5-oxopyrrolidine-2-carboxylate (**PCA dodecyl ester**): Yield 84 % as a white waxy crystals; T_m_ = 44-46 °C; M_w_ = 297.44. ^1^H NMR (500 MHz, CDCl_3_) δ 6.29 (s, 1H), 4.24 (dd, *J* = 8.7, 5.1 Hz, 1H), 4.15 (td, *J* = 6.8, 1.3 Hz, 2H), 2.54 – 2.45 (m, 1H), 2.46 – 2.30 (m, 2H), 2.28 – 2.19 (m, 1H), 1.68 – 1.61 (m, 2H), 1.38 – 1.21 (m, 18H), 0.88 (t, *J* = 6.9 Hz, 3H). ^13^C NMR (126 MHz, CDCl_3_) δ 177.69, 172.00, 65.80, 55.36, 31.87, 29.59, 29.58, 29.52, 29.45, 29.30, 29.18, 29.16, 28.48, 25.77, 24.83, 22.64, 14.07. IR (ATR): ν_max_ 2918; 2849; 1733; 1699; 1258; 1192 cm^-1^. MS (APCI, pos): m/z 298.3 [M+H]^+^.


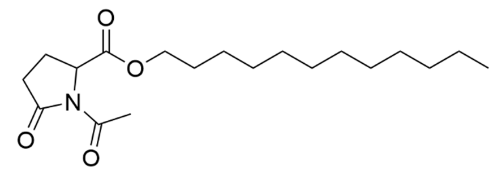


Dodecyl 1-acetyl-5-oxopyrrolidine-2-carboxylate (**PCA2**): Yield 91 % as a colourless oil; M_w_ = 339.48. ^1^H NMR (500 MHz, CDCl_3_) δ 4.75 (dd, *J* = 9.6, 2.6 Hz, 1H), 4.16 (t, *J* = 6.7 Hz, 2H), 2.78 – 2.67 (m, 1H), 2.62 – 2.51 (m, 4H), 2.40 – 2.28 (m, 1H), 2.12 – 2.03 (m, 1H), 1.68 – 1.61 (m, 2H), 1.38 – 1.21 (m, 18H), 0.88 (t, *J* = 6.9 Hz, 3H). ^13^C NMR (126 MHz, CDCl_3_) δ 174.46, 171.07, 170.97, 65.85, 57.84, 31.87, 31.81, 29.60, 29.58, 29.51, 29.46, 29.30, 29.14, 28.45, 25.72, 24.59, 22.65, 21.36, 14.08. IR (ATR): ν_max_ 2924; 2854; 1748; 1704; 1373; 1287; 1192 cm^-1^. MS (APCI, pos): m/z 340.3 [M+H]^+^.


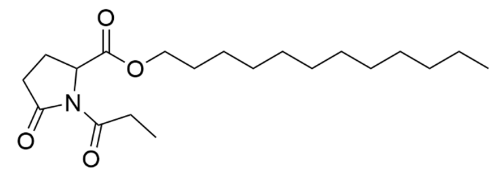


Dodecyl 5-oxo-1-propionylpyrrolidine-2-carboxylate (**PCA3**): Yield 77 % as a colourless oil; M_w_ = 353.50. ^1^H NMR (500 MHz, CDCl_3_) δ 4.76 (dd, *J* = 9.5, 2.6 Hz, 1H), 4.16 (td, *J* = 6.7, 1.3 Hz, 2H), 3.04 – 2.87 (m, 2H), 2.78 – 2.67 (m, 1H), 2.61 – 2.51 (m, 1H), 2.39 – 2.29 (m, 1H), 2.12 – 2.03 (m, 1H), 1.69 – 1.62 (m, 2H), 1.37 – 1.21 (m, 18H), 1.15 (t, *J* = 7.3 Hz, 3H), 0.89 (t, *J* = 6.9 Hz, 3H). ^13^C NMR (126 MHz, CDCl_3_) δ 174.86, 174.37, 171.20, 65.82, 58.01, 31.99, 31.88, 30.25, 29.61, 29.59, 29.53, 29.47, 29.31, 29.16, 28.47, 25.74, 22.66, 21.49, 14.08, 8.06. IR (ATR): ν_max_ 2918; 2853; 1743; 1701; 1366; 1226; 1195 cm^-1^. MS (APCI, pos): m/z 354.3 [M+H]^+^.


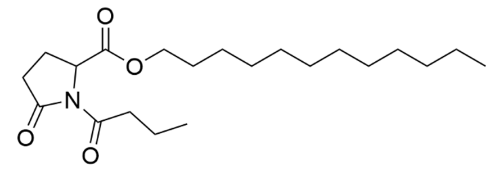


Dodecyl 1-butyryl-5-oxopyrrolidine-2-carboxylate (**PCA4**): Yield 73 % as a colourless oil; M_w_ = 367.53. ^1^H NMR (500 MHz, CDCl_3_) δ 4.76 (dd, *J* = 9.6, 2.7 Hz, 1H), 4.15 (t, *J* = 6.8 Hz, 2H), 3.02 – 2.92 (m, 1H), 2.91 – 2.81 (m, 1H), 2.78 – 2.67 (m, 1H), 2.61 – 2.51 (m, 1H), 2.39 – 2.27 (m, 1H), 2.12 – 2.02 (m, 1H), 1.71 – 1.60 (m, 4H), 1.37 – 1.23 (m, 18H), 0.98 (t, *J* = 7.4 Hz, 3H), 0.89 (t, *J* = 6.9 Hz, 3H). ^13^C NMR (126 MHz, CDCl_3_) δ 174.31, 174.02, 171.19, 65.81, 58.00, 38.47, 32.04, 31.88, 29.61, 29.59, 29.53, 29.47, 29.32, 29.16, 28.48, 25.74, 22.66, 21.43, 17.48, 14.08, 13.63. IR (ATR): ν_max_ 2925; 2854; 1747; 1701; 1281; 1191 cm^-1^. MS (APCI, pos): m/z 368.1 [M+H]^+^.


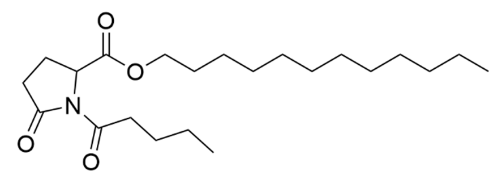


Dodecyl 5-oxo-1-pentanoylpyrrolidine-2-carboxylate (**PCA5**): Yield 73 % as a colourless oil; M_w_ = 381.56. ^1^H NMR (500 MHz, CDCl_3_) δ 4.75 (dd, *J* = 9.5, 2.7 Hz, 1H), 4.15 (t, *J* = 6.7 Hz, 2H), 3.03 – 2.93 (m, 1H), 2.93 – 2.83 (m, 1H), 2.78 – 2.67 (m, 1H), 2.61 – 2.51 (m, 1H), 2.39 – 2.26 (m, 1H), 2.11 – 2.02 (m, 1H), 1.67 – 1.59 (m, 4H), 1.43 – 1.22 (m, 20H), 0.93 (t, *J* = 7.4 Hz, 3H), 0.89 (t, *J* = 6.9 Hz, 3H). ^13^C NMR (126 MHz, CDCl_3_) δ 174.31, 174.20, 171.19, 65.81, 58.03, 36.33, 32.05, 31.88, 29.61, 29.59, 29.53, 29.47, 29.32, 29.17, 28.48, 26.12, 25.75, 22.66, 22.21, 21.42, 14.08, 13.83. IR (ATR): ν_max_ 2925; 2854; 1747; 1701; 1279; 1190 cm^-1^. MS (APCI, pos): m/z 382.1 [M+H]^+^.

### High-performance liquid chromatography (HPLC)

The concentration of model drugs in all samples was measured using the Shimadzu prominence instrument (Shimadzu, Kyoto, Japan) consisting of SIL-20A HT autosampler, LC-20AD pumps with a DGU 20A3 degasser, CTO-20AC column oven, SPD-M20A diode array detector, CBM-20A communication module, and LCsolutions 1.22 software.

TH was quantified on a LiChroCART 250-4 column (LiChrospher 100 RP-18e; 5 µm, Merck, Darmstadt, Germany) at 35°C with the detection at 272 nm. As a mobile phase, 40% methanol in 0.1 M NaH_2_PO_4_ (v/v) was used at a 1.2 ml/min flow rate. The calibration curve was linear in the 0.5 – 100 µg/ml range (r^2^ ≥ 0.999, p < 0.001).^2^

DC was determined on a LiChroCART 250-4 column (LiChrospher 100 RP-18e; 5 µm, Merck, Darmstadt, Germany) at 30°C using a mobile phase consisting of acetonitrile, water, and acetic acid in a ratio 90:60:5 at the flow rate of 2 ml/min; DC was monitored at 275 nm. The calibration curve was linear in the range of 0.5 – 100 µg/ml (r^2^ ≥ 0.999, p < 0.001). The lower limit of quantification was defined as the lowest amount of an analyte that can be quantitatively determined with ± 20% precision and accuracy.^3^

### Cellular toxicity assessment

The inherent toxicities of selected enhancers were assessed by their IC_50_ (concentration of enhancer, which leads to a 50% decrease in the cell viability) determined *in vitro* on two different non-malignant cell lines, 3T3 mouse fibroblasts (American Type Culture Collection, ATCC, Manassas, USA), and HaCaT spontaneously immortalized human keratinocytes (Cell Lines Service, Eppelheim, Germany). Both cell lines were cultivated in Dulbecco’s modified Eagle’s medium (DMEM, Lonza, Basel, Switzerland) supplemented with 10% heat-inactivated fetal bovine serum (Merck, Darmstadt, Germany), 10 mM HEPES buffer (Merck, Darmstadt, Germany), and 1% penicillin/streptomycin solution (Lonza, Basel, Switzerland) in 75-cm^2^ tissue culture flasks (TPP, Trasadingen, Switzerland) at 37°C in a humidified atmosphere of 5% CO_2_. Subconfluent cells were sub-cultured every 3‑4 days.

For toxicity studies, the stock‑solutions of studied compounds were prepared in dimethyl sulfoxide (DMSO). These stock samples were diluted to the required concentration by the cell culture medium immediately before adding them onto the cells. The amount of DMSO applied to the cells was kept under its intrinsic measurable toxicity limit (below 1 vol.%). The cells were seeded at 10,000 cells per well into 96‑well plates (TPP, Trasadingen, Switzerland).

After 48‑h incubation with the studied enhancer, cell viability was determined *via* 3‑(4,5‑dimethylthiazol‑2‑yl)‑2,5‑diphenyltetrazolium bromide (MTT) uptake assay based on the transformation of yellow MTT into purple formazan by mitochondrial reductase activity of living cells. A solution of MTT in PBS was prepared (3 mg/ml) and added to each well of 96-well plates incubated with a studied enhancer (25 µl per well). After 2‑h incubation at 37°C, the medium with MTT was completely removed, and the cells underwent lysis (100 µl of DMSO applied into each well, followed by vigorous shaking at rt for 12 h). Afterward, the cell viability was assessed by optical density measurement using a Tecan Infinite 200 M plate reader (Tecan, Grödig, Austria) at λ = 570 nm.

Another approach used for the toxicity assessment was the neutral red (NR) uptake assay. NR is a neutral molecule that can easily cross biological membranes. In the acidic environment of lysosomes in living cells, NR becomes charged and accumulates inside lysosomes. After 2‑h incubation with NR (applied in cultivation medium, 40 µg/ml, 100 µl per well), the cells were fixed in 1% CaCl_2_ in 0.5% formaldehyde (100 µl per well) for 15 min. This procedure was followed by two washing steps with PBS (100 µl per well at each step) and the lysis of the cells using 1% acetic acid in 50% ethanol (200 µl per well). The plates were shaken at rt for 30 min, and the optical density of the soluble NR was measured at λ = 540 nm using a Tecan Infinite 200 M plate reader.

The viability of each experimental group was expressed as the percentage of the untreated controls incubated under the same conditions (100%).

### Laser scanning confocal microscopy

Laser scanning confocal microscopy was employed in the inspection of morphological changes of the cells (3T3 and HaCaT). The cells were seeded at a density of 70,000 cells *per* well into 4‑well cell‑imaging slides (Eppendorf, Hamburg, Germany) and incubated under the same conditions as in the toxicity studies using the enhancer concentration corresponding to their IC_15_ and IC_85_. After 48‑h incubation, cells were rinsed twice with warm PBS and fixed using 4% paraformaldehyde in the cell-culture medium for 15 min at 37°C. All subsequent steps are performed at rt. After fixation, the samples were washed three times with PBS, permeabilized for 15 min (0.5% Triton X-100 in PBS), washed three times, and blocked for 60 min (3% bovine serum albumin in PBS). Staining for microfilaments and microtubules was performed using 5 U/ml Alexa Fluor 555 phalloidin (Molecular Probes, Eugene, USA) and 2 µg/ml α‑tubulin antibody, Alexa Fluor 488 conjugate (Thermo Fisher Scientific, Waltham, USA), respectively, for 90 min. Specimens were rinsed twice with PBS and stained with 4 nM Hoechst 33342 in PBS for 10 min. Removing excess salts from samples was performed by washing with demineralized water for 5 min. Dry samples were mounted on a glass slide with ProLong Gold antifade mountant (Molecular Probes, Eugene, USA) overnight at room temperature and sealed using colorless nail polish. A Nikon A1+ confocal system (Nikon, Tokyo, Japan) equipped with NIS Elements AR 4.20 software (Laboratory Imaging, Praha, Czech Republic) was employed to visualize all structures using a 20× objective lens and 405, 488, and 561 nm lasers. Eight focal planes (pinhole diameter = 19.16 μm) were taken to cover the whole volume of samples.

## Figure S1


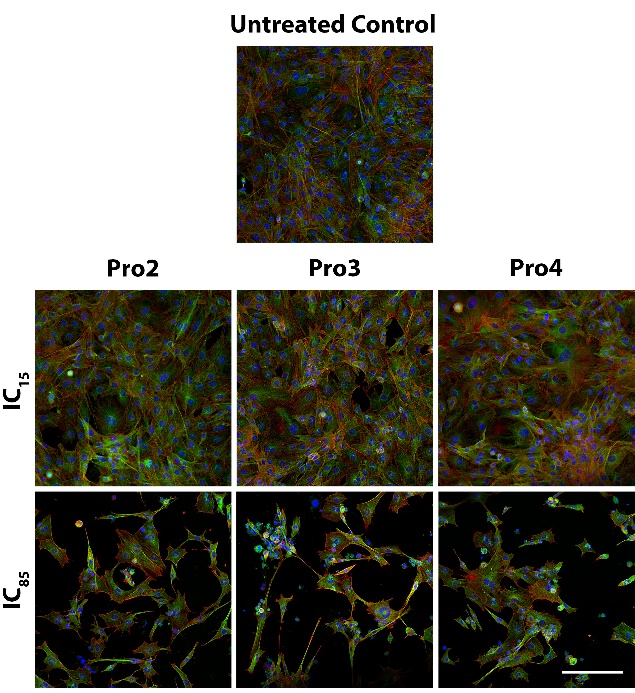

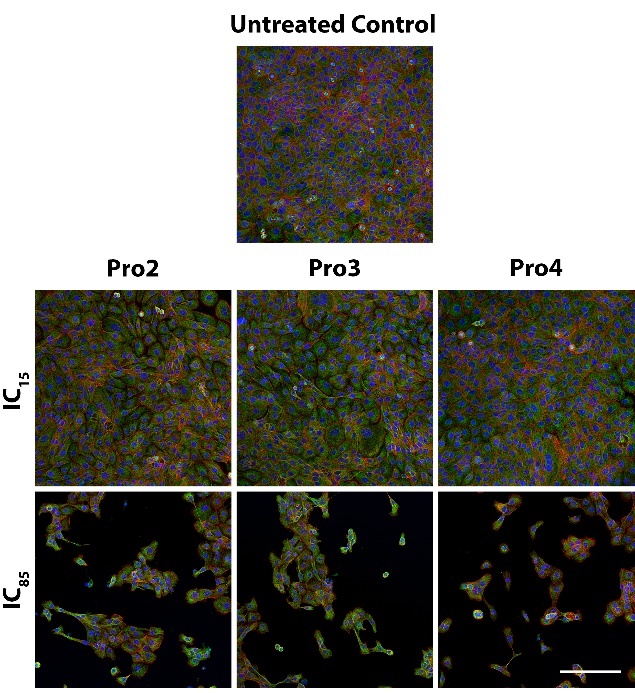


**Figure S1.** Confocal images of 3T3 (left panel) and HaCaT (right panel) cells, untreated (control) or treated with studied enhancers Pro2, Pro3, and Pro4 in a concentration corresponding to their IC_15_ and IC_85_ values for 48 h. Cells were stained for nuclei (blue), microfilaments (green), and microtubules (red). The bar represents 200 µm.

## Table S1

**Table S1.** TEWL and impedance values before the experiment and after treatment of skin with 60% PG without (control) or with 1% proline derivatives, 1% Azone or 5% sodium dodecyl sulfate (SDS).

| **compd.** | **before** | **after the sample had been removed** | | | | |
| --- | --- | --- | --- | --- | --- | --- |
|  |  | **1 h** | **4 h** | **8 h** | **12 h** | **24 h** |
| **TEWL (g/m^2^/h ± SEM)** | | | | | | |
| **control** | 24.7 ± 1.5 | 33.2 ± 0.6 | 33.3 ± 1.6 | 29.0 ± 2.6 | 26.2 ± 2.4 | 20.5 ± 1.0^+^ |
| **Pro2** | 25.0 ± 3.0 | 65.4 ± 1.5*^≠^ | 56.0 ± 1.3*^≠^ | 45.9 ± 1.5*^≠+^ | 42.5 ± 2.5*^≠+^ | 41.8 ± 1.0*^≠+^ |
| **Pro3** | 27.5 ± 6.3 | 59.4 ± 5.6*^≠^ | 47.1 ± 1.7*^≠+^ | 36.5 ± 1.1^+^ | 30.2 ± 2.2^+^ | 27.4 ± 2.2^+^ |
| **Pro4** | 27.3 ± 4.1 | 49.9 ± 5.2*^≠^ | 39.4 ± 2.5*^+^ | 30.5 ± 1.3^+^ | 26.3 ± 1.8^+^ | 25.0 ± 2.8^+^ |
| **Azone** | 28.2 ± 3.6 | 67.7 ± 1.3*^≠^ | 60.7 ± 1.7*^≠^ | 51.9 ± 1.6*^≠+^ | 48.8 ± 2.3*^≠+^ | 48.3 ± 1.4*^≠+^ |
| **SDS** | 26.1 ± 2.9 | 47.3 ± 2.2*^≠^ | 45.8 ± 4.4* | 38.7 ± 3.1* | 38.2 ± 2.6* | 38.0 ± 2.3* |
| **impedance (kΩ×cm^2^ ± SEM)** | | | | | | |
| **control** | 28.2 ± 1.9 | 5.4 ± 1.1* | 13.5 ± 0.3*^+^ | 21.7 ± 0.8*^+^ | 24.3 ± 1.0^+^ | 27.1 ± 0.9^+^ |
| **Pro2** | 25.2 ± 2.1 | 2.8 ± 0.1* | 6.0 ± 1.1*^≠^ | 8.1 ± 1.7*^≠^ | 8.6 ± 2.0*^≠^ | 9.4 ± 2.6*^≠+^ |
| **Pro3** | 28.9 ± 1.7 | 1.4 ± 0.8* | 7.9 ± 0.4*^≠+^ | 13.1 ± 0.7*^≠+^ | 15.8 ± 1.0*^≠+^ | 18.6 ± 0.9*^≠+^ |
| **Pro4** | 28.9 ± 2.6 | 3.8 ± 0.8* | 11.5 ± 1.7*^+^ | 18.3 ± 2.5*^+^ | 21.2 ± 2.5*^+^ | 23.8 ± 2.9^+^ |
| **Azone** | 25.1 ± 2.0 | 2.6 ± 0.4* | 7.8 ± 0.4*^≠^ | 11.2 ± 0.2*^≠+^ | 12.2 ± 0.4*^≠+^ | 12.4 ± 0.8*^≠+^ |
| **SDS** | 26.7 ± 0.7 | 1.9 ± 0.2* | 3.8 ± 0.2*^≠^ | 5.0 ± 0.3*^≠^ | 5.6 ± 0.3*^≠^ | 6.4 ± 0.3*^≠^ |

Samples were removed from the skin after 24 h, and TEWL and impedance values were recorded in predetermined time intervals (1, 4, 8, 12, and 24 h after sample removal). Data are presented as the means ± SEM; n ≥ 3. * statistically significant difference compared to the baseline value before the sample application at p < 0.05. ^+^ statistically significant compared to the value 1 h after the sample removal at p < 0.05. ^≠^ statistically significant compared to the control (60% PG without enhancer) at the same time point, at p < 0.05.

## References

1 Janůšová, B. *et al.* Amino acid derivatives as transdermal permeation enhancers. *J Control Release* **165**, 91-100 (2013).

2 Novotný, J., Janůšová, B., Novotný, M., Hrabálek, A. & Vávrová, K. Short-chain ceramides decrease skin barrier properties. *Skin Pharmacol Physiol* **22**, 22-30 (2008).

3 FDA. Bioanalytical method validation guidance for industry. 1-41 (2018).
